# 

**Published:** 1857

**Authors:** 


AN INDEPENDENT JOURNAL,

DEVOTED TO

DENTAL PROGRESS

AND

IMPROVEMENTS IN THE MANUFACTURE AND USE OF INSTRUMENTS AND MATERIALS.

COMPILED BY JNO. T. TOLAND,

ISSUED QUARTERLY.

PUBLISHED BY

JNO. T. TOLAND,

WHOLESALE DEALER IN TEETH AND PLATE, INSTRUMENTS, FILES, AND TOOLS, AND EVERY DESCRIPTION OF DENTAL MATERIALS.

No. 38 West Fourth St., Cincinnati, O.

1857.

